# Author Correction: Hurricane María tripled stem breaks and doubled tree mortality relative to other major storms

**DOI:** 10.1038/s41467-019-10159-3

**Published:** 2019-05-01

**Authors:** María Uriarte, Jill Thompson, Jess K. Zimmerman

**Affiliations:** 1 Department of Ecology Evolution and Environmental Biology, Columbia University, 1200 Amsterdam Avenue, New York, NY 10027 USA; 2 Centre for Ecology & Hydrology Bush Estate, Penicuik, Midlothian EH26 0QB UK; 3 Department of Environmental Sciences, University of Puerto Rico, San Juan, Puerto Rico 00925 USA

**Keywords:** Climate-change ecology, Community ecology, Forest ecology

Correction to: *Nature Communications* 10.1038/s41467-019-09319-2, published online 25 March 2019.

The original version of this Article omitted the following from the Acknowledgements:

Gabriel Arellano was instrumental in collecting post-María Hurricane data, as part of the Next Generation Ecosystem Experiments-Tropics, funded by the U.S. Department of Energy, Office of Science, Office of Biological and Environmental Research.

This has now been corrected in both the PDF and HTML versions of the Article.

