# Supplementary material for: Hurricane María tripled stem breaks and doubled tree mortality relative to other major storms
Source: Nat Commun. 2019 Mar 25;10:1362. doi: 10.1038/s41467-019-09319-2 (PMC6433954; doi:10.1038/s41467-019-09319-2)
Supplement: Supplementary file 1 — Supplementary information [file 41467_2019_9319_MOESM1_ESM.pdf]

HURRICANE MARÍA TRIPLED STEM BREAKS AND DOUBLED TREE MORTALITY RELATIVE TO OTHER MAJOR STORMS

Uriarte et al.

SUPPLEMENTARY MATERIALS

Supplementary Table 1. Number of stems with dbh ≥ 10 cm that were assessed for damage after Hurricanes Hugo, Georges, and María. For context on forest composition at the time Georges struck, we provide number of stems ≥ 10 cm in the 1995 LFDP census.

Species	Family	Code	No. of stems in Hugo	No. stems in Georges (1995 census)	No. of stems in Maria
Alchornea floribunda	Euphorbiaceae	ALCFLO	92	15 (79)	75
Alchornea latifolia	Euphorbiaceae	ALCLAT	204	27 (188)	132
Andira inermis	Leguminosae	ANDINE	73	17 (81)	86
Buchenavia capitata	Combretaceae	BUCCAP	190	34 (175)	154
Byrsonima spicata	Malpighiaceae	BYRSPI	155	31 (147)	94
Casearia arborea	Salicaceae	CASARB	1,053	167 (1,014)	782
Casearia sylvestris	Salicaceae	CASSYL	208	44 (201)	59
Cecropia schreberiana	Moraceae	CECSCH	163	248 (1,269)	818
Cordia sulcata	Boraginaceae	CORSUL	145	28 (149)	100
Dacryodes excelsa	Burseraceae	DACEXC	1,023	201 (1,079)	1,135
Drypetes glauca	Euphorbiaceae	DRYGLA	157	32 (152)	104
Guarea guidonia	Meliaceae	GUAGUI	306	59 (315)	272
Guettarda valenzuelana	Rubiaceae	GUEVAL	113	25 (107)	67
Homalium racemosum	Salicaceae	HOMRAC	179	23 (178)	151
Inga laurina	Leguminosae	INGLAU	557	83 (498)	349
Laetia procera	Salicaceae	LAEPRO	54	6 (56)	74
Manilkara bidentata	Sapotaceae	MANBID	670	142 (694)	749
Matayba dominguensis	Sapindaceae	MATDOM	269	56 (251)	152
Ocotea leucoxylon	Lauraceae	OCOLEU	164	34 (163)	96
Prestoea acuminata	Palmae	PREACU	4,415	1,201 (7,401)	8,151
Schefflera morototoni	Araliaceae	SCHMOR	202	46 (214)	480
Sloanea berteriana	Elaeocarpaceae	SLOBER	508	127 (542)	454
Tabebuia heterophylla	Bignoniaceae	TABHET	330	65 (317)	148
Tetragastris balsamifera	Burseraceae	TETBAL	125	24 (126)	146

Supplementary Table 2. Percent of stems broken, uprooted, and immediately killed for the study species in Hugo, Georges, and María. Note that for Georges immediate mortality was not assessed and stem break and uprooting rates are based on the subset of stems assessed for damage (see methods for details).

Damage	Hugo	Georges	María
% stem break	8.45	4.55	17.46
% uprooting	6.69	1.54	6.80
% immediate mortality	7.71	NA	15.40

Supplementary Table 3. Percent of stems broken, uprooted, and immediately killed for study species in Hugo, Georges, and Maria. Annual rates over a period (2011-2016) free of severe storms (NSS) are also shown. For Georges we only provide rates for species with ≥40 stems assessed for damage.

Species code	% break Hugo	% break Georges	% break Maria	% break NSS.	% Uproot. Hugo	% Uproot. Georges	% Uproot. Maria	% Uproot. Georges	% Uproot NSS	% mortality Hugo	% mortality Maria	% mortality NSS.
ALCFLO	13.04		40.00	0.88	25.00		17.00		0.88	11.9	8.01	0.29
ALCLAT	12.25		44.00	3.55	7.35		8.00		0.24	4.41	10.61	0.71
ANDINE	5.48		40.00	0.47	2.74		0.00		0.00	0	6.97	0.00
BUCCAP	4.21		32.00	1.29	13.16		13.00		0.29	0.5	15.19	0.14
BYRSPI	5.81		28.00	0.42	9.68		12.00		0.63	7.74	18.08	0.21
CASARB	10.45	11.38	23.00	2.55	20.23	4.19	14.00	4.19	0.34	8.73	17.52	1.15
CASSYL	9.62	0.00	20.00	4.87	8.65	0.00	6.00		0.00	0	11.86	1.39
CECSCH	33.08	6.85	30.00	4.17	17.69	3.23	25.00	3.23	0.51	40.49	55.13	2.42
CORSUL	8.28		24.00	2.06	0.00		6.00		0.00	2.06	4	0.37
DACEXC	4.30	5.47	19.00	0.20	1.08	0.00	3.00	0.00	0.02	1.46	5.02	0.11
DRYGLA	5.73		27.00	1.95	8.92		13.00		0.33	4.45	14.42	1.46
GUAGUI	7.19	5.08	38.00	1.05	6.86	1.69	4.00	1.69	0.00	1.31	2.94	0.90
GUEVAL	8.85		27.00	1.39	6.19		11.00		0.00	3.53	14.92	1.11
HOMRAC	2.79		34.00	1.74	3.35		3.00		0.25	2.23	4.63	0.50
INGLAU	15.98	16.87	27.00	1.56	8.26	1.20	8.00	1.20	0.23	12.92	10.03	0.40
LAEPRO	5.56		36.00	0.82	0.00		8.00		0.00	3.71	18.92	1.10
MANBID	6.27	0.70	23.00	0.32	3.58	0.00	4.00	0.00	0.03	1.64	3.47	0.11
MATDOM	3.72	5.35	39.00	1.45	8.55	0.00	9.00		0.12	7.06	17.76	0.48
OCOLEU	13.41		32.00	3.90	12.80		12.00		0.12	1.82	17.71	0.85
PREACU	5.64	0.75	5.00	0.47	1.11	0.42	2.00	0.42	0.02	7.1	14.67	0.60
SCHMOR	14.36	10.87	31.00	1.01	7.43	4.35	13.00		0.16	14.85	25	1.21
SLOBER	10.24	6.55	29.00	1.47	4.13	0.00	8.00	4.35	0.12	3.54	9.03	1.06
TABHET	6.67	7.69	37.00	3.14	2.42	1.54	1.00	1.54	0.21	5.15	22.3	1.88

TETBAL	2.40		20.00	0.28	6.40		5.00		0.14	3.2	4.11	0.28
Average	**8.97**	**6.56**	**29.38**	**1.71**	**7.73**	**1.51**	**8.54**	**1.85**	**0.19**	**6.24**	**13.85**	**0.78**

Supplementary Table 4. Maximum dbh (cm) at the time each storm struck the forest and mean wood density (g. cm^{-3}) for the 24 study species.

Species code	Hugo max dbh	Georges max dbh	Maria max dbh	Wood density
ALCFLO	75.8	78.5	54	0.43
ALCLAT	66	64	72	0.40
ANDINE	55.1	57.2	58	0.65
BUCCAP	150.9	151.5	160	0.64
BYRSPI	68	69.4	78	0.61
CASARB	48.7	43.3	36	0.58
CASSYL	27.2	25.7	16	0.71
CECSCH	48	47.6	56	0.26
CORSUL	33.5	32.9	34	0.61
DACEXC	82.2	82.4	88	0.53
DRYGLA	38.2	39.7	40	0.67
GUAGUI	96.3	99.3	107	0.59
GUEVAL	65.7	66.2	42	0.60
HOMRAC	110.5	118	103	0.79
INGLAU	87	81.1	78	0.63
LAEPRO	50.2	50.5	47	0.64
MANBID	78.7	80	90	0.86
MATDOM	59	60.5	64	0.69
OCOLEU	52	51.1	56	0.46
PREACU	42	36.7	23	0.31
SCHMOR	75	66.1	53	0.42
SLOBER	93.3	89.3	94	0.77
TABHET	69.2	69.9	64	0.66
TETBAL	51.8	53.5	61	0.53

Supplementary Table 5. Average estimated probability of breakage, uprooting, and mortality for the study species in Hugo, Georges, and María after accounting for effects of diameter and exposure to wind. Values represent random species effects for mixed models that incorporate diameter and wind exposure.

Species	Mortality Maria	Break Maria	Uprooting Maria	Mortality Hugo	Break Hugo	Uprooting Hugo
ALCFLO	0.09	0.42	0.21	0.11	0.12	0.23
ALCLAT	0.11	0.45	0.10	0.05	0.12	0.07
ANDINE	0.08	0.39	0.02	0.02	0.06	0.03
BUCCAP	0.06	0.39	0.20	0.02	0.05	0.12
BYRSPI	0.17	0.31	0.14	0.08	0.06	0.09
CASARB	0.17	0.28	0.21	0.10	0.10	0.20
CASSYL	0.12	0.37	0.11	0.01	0.09	0.08
CECSCH	0.55	0.34	0.29	0.50	0.30	0.16
CORSUL	0.05	0.23	0.06	0.02	0.08	0.01
DACEXC	0.05	0.19	0.03	0.02	0.04	0.01
DRYGLA	0.14	0.27	0.12	0.05	0.06	0.08
GUAGUI	0.04	0.42	0.05	0.02	0.07	0.06
GUEVAL	0.14	0.28	0.11	0.04	0.09	0.06
HOMRAC	0.05	0.34	0.04	0.02	0.04	0.03
INGLAU	0.10	0.31	0.12	0.13	0.16	0.08
LAEPRO	0.18	0.37	0.09	0.04	0.07	0.02
MANBID	0.04	0.25	0.05	0.02	0.06	0.03
MATDOM	0.17	0.40	0.10	0.08	0.04	0.08
OCOLEU	0.17	0.40	0.16	0.03	0.13	0.12
PREACU	0.15	0.05	0.02	0.07	0.06	0.01
SCHMOR	0.25	0.34	0.17	0.16	0.14	0.07
SLOBER	0.09	0.34	0.11	0.05	0.10	0.04
TABHET	0.22	0.38	0.04	0.06	0.07	0.03
TETBAL	0.05	0.24	0.06	0.04	0.04	0.06

Supplementary Table 6. Effects of stem diameter on the probability of stem break, uprooting, or immediate mortality in H. Hugo and María. Diameter was scaled prior to analyses so the coefficient represent the change in rates with one standard deviation change in diameter. * indicates that the effect was significant at p=0.05. See Fig. 4 for species-specific relationships between stem size and damage and mortality.

Process	Hugo	Maria
Stem break	0.09 (0.09)	0.21 (0.06)*
Uprooting	0.77 (0.11)*	0.41 (0.09)*
Immediate mortality	1.20 (0.11)*	0.14 (0.08)

Supplementary Table 7. R^2 and significance of linear regression models of rates of mortality, uprooting and breakage as a function of wood density during Hurricanes Hugo and María as well as average annual rates in a storm-free period (2011-2016). *indicates relationship is significant at p=0.10.

Process	Conditions	R^2	*p*
% Mortality			
	Hugo*	0.32	0.002
	Georges	NA	NA
	Maria*	0.23	0.01
	No severe storm	0.02	0.23
% Stem break			
	Hugo*	0.29	0.003
	Georges	-0.08	0.74
	Maria	-0.03	0.64
	No severe storm	0.01	0.41
% Uprooting			
	Hugo*	0.09	0.09
	Georges	0.16	0.12
	Maria*	0.16	0.02
	No severe storms	0.04	0.16

Supplementary Table 8. Number of stems for the 24 study species that were broken, uprooted or undamaged in Hugo and proportion of delayed mortality recorded in 1995.

Damage in Hugo 1989	No. stems alive 1995	No. stems dead 1995	Prop. delayed mortality
Broken	269	115	0.30
Uprooted	171	213	0.55
Undamaged	9,089	571	0.06

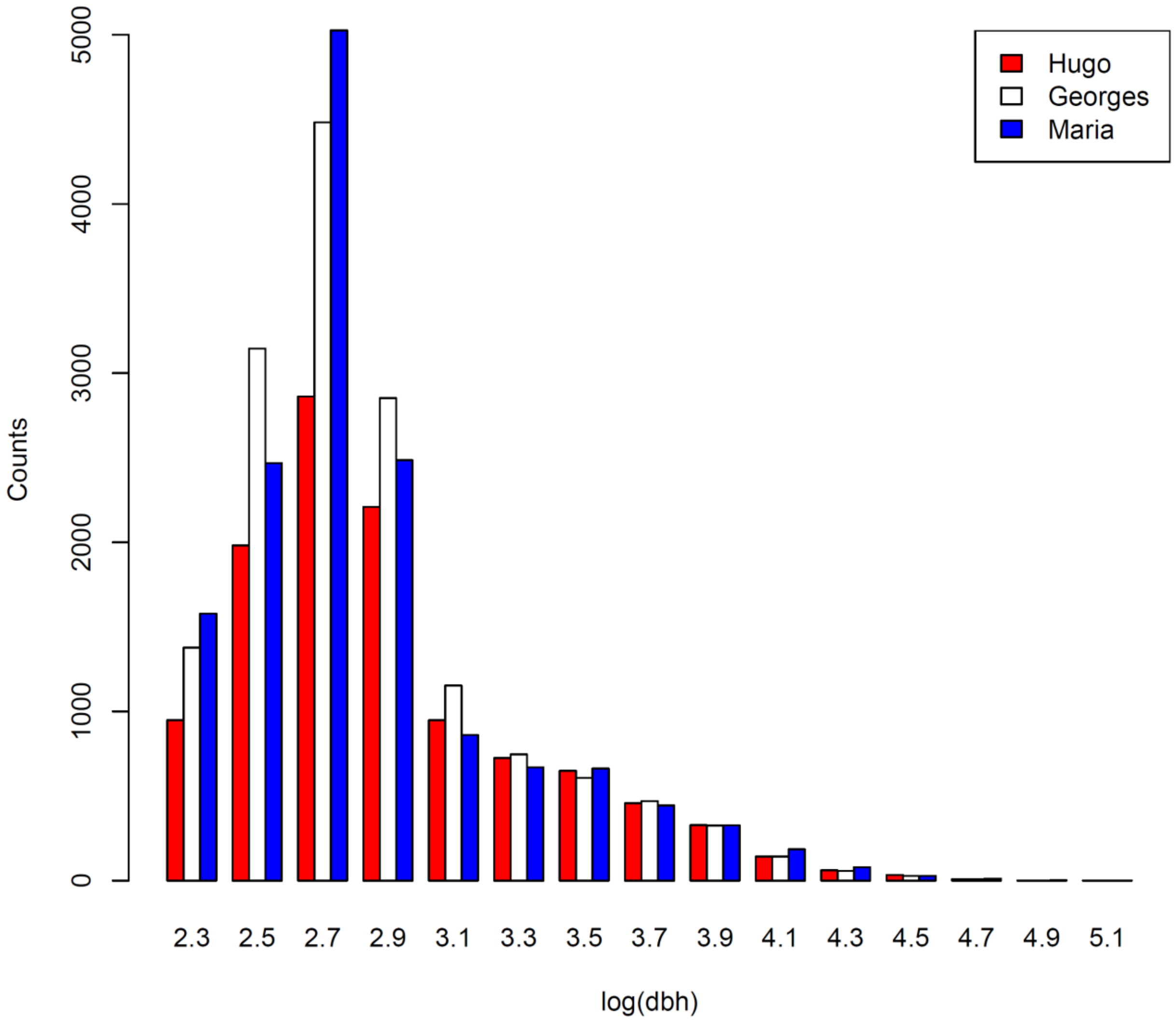

Supplementary Figure 1. Diameter size distributions for all stems ≥10 cm dbh in the LFDP at the time Hurricanes Hugo (red bars), Georges (white bars), and María (blue bars) struck the site. Chi-square tests for differences in the size distributions among any two storms were not significant at $p=0.05$.

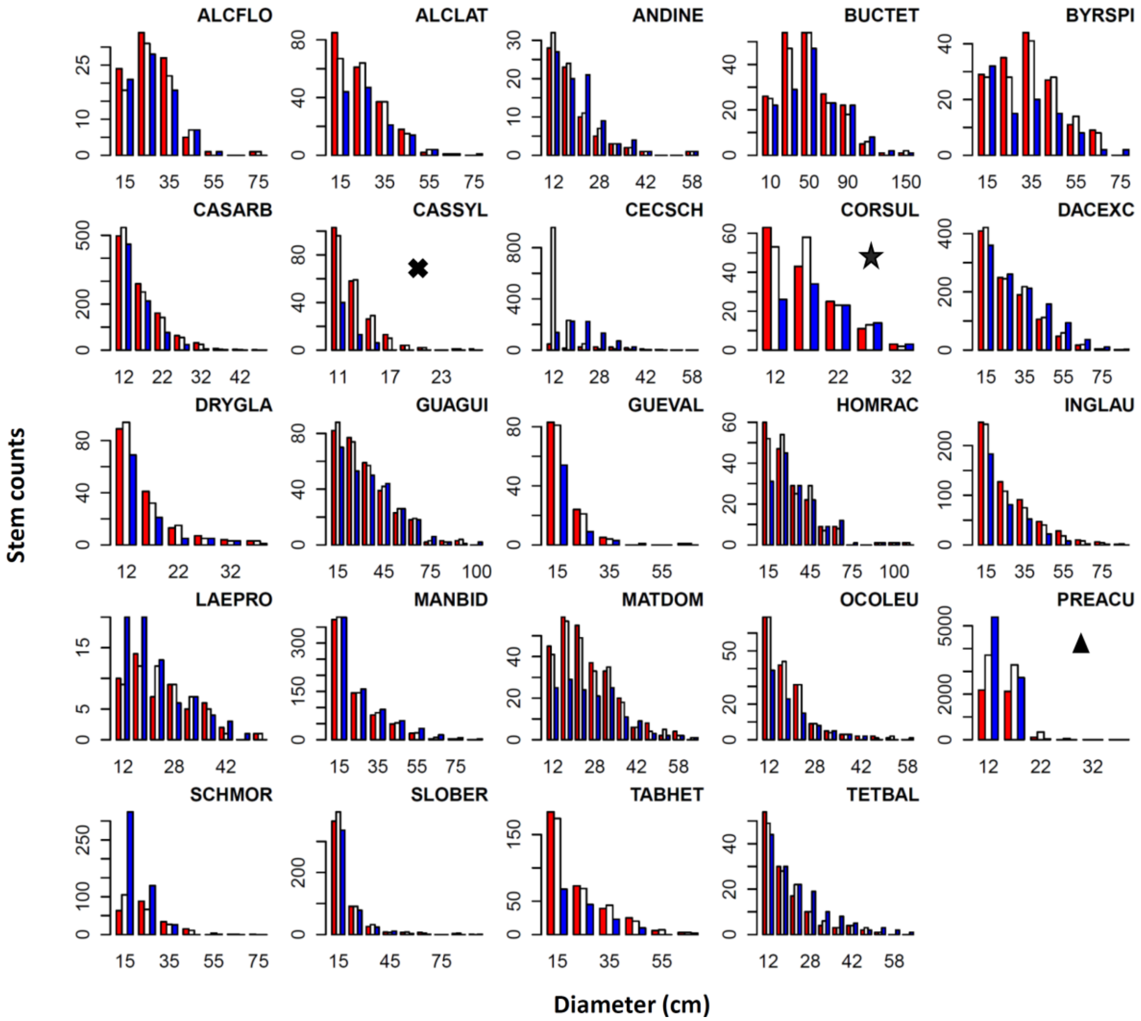

Supplementary Figure 2. Diameter size distributions at the time Hurricanes Hugo (red bars), Georges (white bars), and Maria (blue bars) struck the site for the 24 species included in the study. Six letter codes for species are provided in Table S1. A star indicate that χ^2 tests indicated that size distributions among all two pairs of the three storms were significantly different at p=0.05. A cross indicates that size distributions between Georges and Hugo and a triangle between Hugo and María differed.

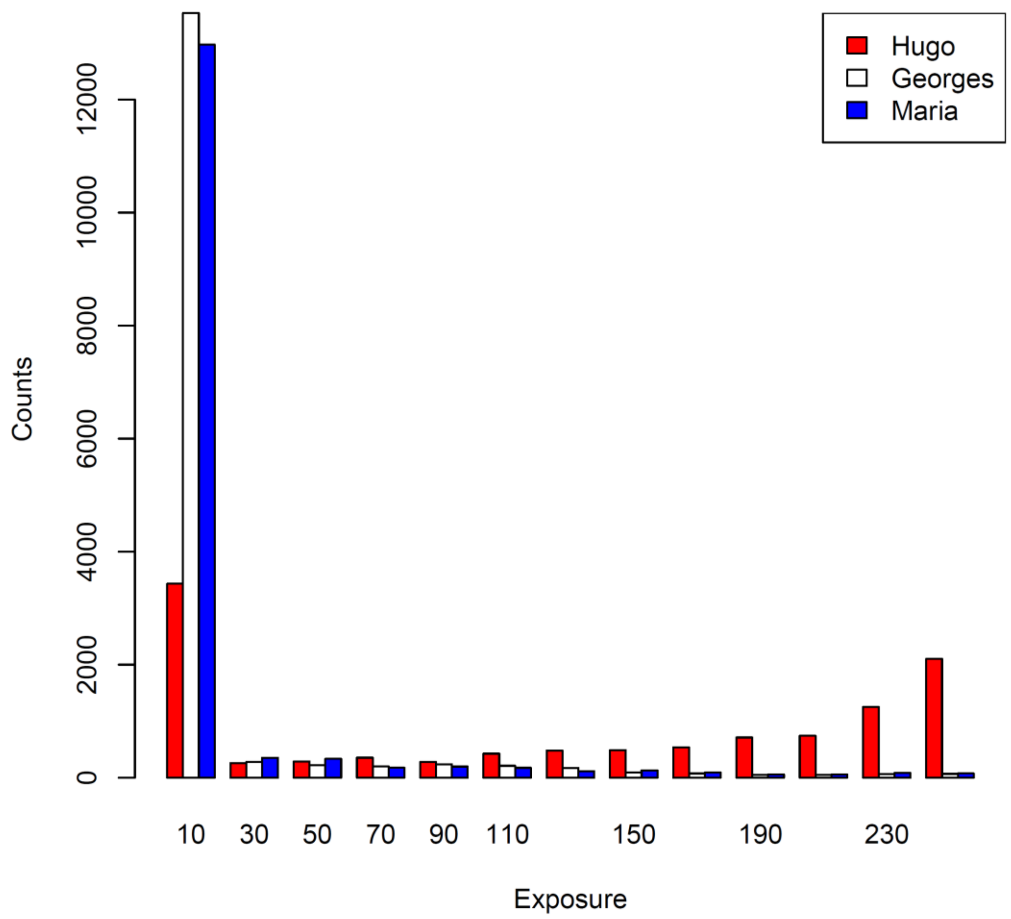

Supplementary Figure 3. Distribution of wind exposure for all stems of 24 study species during Hurricanes Hugo (red bars), Georges (white bars), and María (blue bars). The exposure index is 0 = no exposure and 255 = high exposure. The main reason for differences in exposure between Hugo and the other two storms is the track of the storms (Fig. 1A). Despite the fact that the maximum wind speed recorded for Maria was higher than Hugo or Georges, Hurricane Maria, like Georges, passed to the south side of the Luquillo Mountains, which protected the LFDP forest area on the northern slopes of the mountains from some of the wind force. Hurricane Hugo passed to the North of the Luquillo Mountains so the forest suffered greater exposure to this storm.
