# Supplementary material for: Hurricane María tripled stem breaks and doubled tree mortality relative to other major storms
Source: Nat Commun. 2019 Mar 25;10:1362. doi: 10.1038/s41467-019-09319-2 (PMC6433954; doi:10.1038/s41467-019-09319-2)
Supplement: Supplementary file 3 — Reporting Summary [file 41467_2019_9319_MOESM3_ESM.pdf]

nature research

Corresponding author(s): *Double-blind peer review submissions: write DBPR and your manuscript number here instead of author names.*

Reporting Summary

Nature Research wishes to improve the reproducibility of the work that we publish. This form provides structure for consistency and transparency in reporting. For further information on Nature Research policies, see Authors & Referees and the Editorial Policy Checklist.

Statistical parameters

When statistical analyses are reported, confirm that the following items are present in the relevant location (e.g. figure legend, table legend, main text, or Methods section).

n/a	Confirmed	
☐	☒	The exact sample size (*n*) for each experimental group/condition, given as a discrete number and unit of measurement
☐	☒	An indication of whether measurements were taken from distinct samples or whether the same sample was measured repeatedly
☐	☒	The statistical test(s) used AND whether they are one- or two-sided<br>*Only common tests should be described solely by name; describe more complex techniques in the Methods section.*
☐	☒	A description of all covariates tested
☐	☒	A description of any assumptions or corrections, such as tests of normality and adjustment for multiple comparisons
☐	☒	A full description of the statistics including central tendency (e.g. means) or other basic estimates (e.g. regression coefficient) AND variation (e.g. standard deviation) or associated estimates of uncertainty (e.g. confidence intervals)
☐	☒	For null hypothesis testing, the test statistic (e.g. *F*, *t*, *r*) with confidence intervals, effect sizes, degrees of freedom and *P* value noted<br>*Give P values as exact values whenever suitable.*
☒	☐	For Bayesian analysis, information on the choice of priors and Markov chain Monte Carlo settings
☐	☒	For hierarchical and complex designs, identification of the appropriate level for tests and full reporting of outcomes
☒	☐	Estimates of effect sizes (e.g. Cohen's *d*, Pearson's *r*), indicating how they were calculated
☒	☐	Clearly defined error bars<br>*State explicitly what error bars represent (e.g. SD, SE, CI)*

Our web collection on statistics for biologists may be useful.

Software and code

Policy information about availability of computer code

Data collection	We used our R code. It is available upon request.
Data analysis	R statistical software; base packages and lme4.

For manuscripts utilizing custom algorithms or software that are central to the research but not yet described in published literature, software must be made available to editors/reviewers upon request. We strongly encourage code deposition in a community repository (e.g. GitHub). See the Nature Research guidelines for submitting code & software for further information.

Data

Policy information about availability of data

All manuscripts must include a data availability statement. This statement should provide the following information, where applicable:

- Accession codes, unique identifiers, or web links for publicly available datasets
- A list of figures that have associated raw data
- A description of any restrictions on data availability

Data for the survival analyses and damage data for Hurricane Hugo is available in the Luquillo LTER data repository (https://luq.lter.network/datacatalog). Tree damage data for Maria will be available at the species level.

Field-specific reporting

Please select the best fit for your research. If you are not sure, read the appropriate sections before making your selection.

☐ Life sciences ☐ Behavioural & social sciences ☒ Ecological, evolutionary & environmental sciences

For a reference copy of the document with all sections, see nature.com/authors/policies/ReportingSummary-flat.pdf

Ecological, evolutionary & environmental sciences study design

All studies must disclose on these points even when the disclosure is negative.

Study description	We sampled hurricane damage for all trees >= 10cm diameter after two severe storms and mortality over a 5 year period following the first storm (Hurricane Hugo) and a subset of the plot for Hurricane Georges. Data was collected for all trees in a 16 ha plot in Hugo and Maria and for a subset of trees for Georges.
Research sample	We sampled all tree species that had at least 50 stems in each storm. Sample sizes for each species are provided in Supp. Table 1.
Sampling strategy	We sampled all trees >=10cm dbh since this size class suffers the brunt of the damage.
Data collection	Data collection protocols follows standardized protocols from Smithsonian ForestGeo and the Luquillo LTER.
Timing and spatial scale	First damage assessment occurred in 1990 within a year after Hurricane Hugo (1989) and Georges (1998). Damage data for Hurricane Maria was collected between January and May of 2018. All trees >=10cm in diameter were sampled in a 16 hectare plot.
Data exclusions	We excluded species with fewer than 50 stems in any of the damage assessment because a small number of stems would preclude robust comparisons between the two storms.
Reproducibility	This is an observational study after a natural disaster. Data collection protocols were detailed. Data was entered twice and any discrepancies were resolved in the field.
Randomization	NA
Blinding	NA

Did the study involve field work? ☒ Yes ☐ No

Field work, collection and transport

Field conditions	This is a subtropical forest. Rainfall averages 3500mm per year and temperature is relatively constant throughout the year, ranging between 23 and 27 deg C.
Location	The Luquillo Forest Dynamics Plot in El Yunque National Forest, Puerto Rico.
Access and import/export	NA
Disturbance	Our sampling had a minor effect on the forest.

Reporting for specific materials, systems and methods

Materials & experimental systems

n/a	Involved in the study
☒	☐ Unique biological materials
☒	☐ Antibodies
☒	☐ Eukaryotic cell lines
☒	☐ Palaeontology
☒	☐ Animals and other organisms
☒	☐ Human research participants

Methods

n/a	Involved in the study
☒	☐ ChIP-seq
☒	☐ Flow cytometry
☒	☐ MRI-based neuroimaging
